# Developing an analytical tool for evaluating EMS system design changes and their impact on cardiac arrest outcomes: combining geographic information systems with register data on survival rates

**DOI:** 10.1186/1757-7241-21-8

**Published:** 2013-02-15

**Authors:** Björn Sund

**Affiliations:** 1 Swedish Civil Contingencies Agency (MSB), Karlstad, Sweden; 2 Department of Economics and Statistics, Karlstad Business School, Karlstad University, Karlstad, Sweden; 3 Swedish Business School, Örebro University, Örebro, Sweden

**Keywords:** Out-of-hospital cardiac arrest, Defibrillation, Response time, Survival rate, Geographic information systems, Fire services

## Abstract

**Background:**

Out-of-hospital cardiac arrest (OHCA) is a frequent and acute medical condition that requires immediate care. We estimate survival rates from OHCA in the area of Stockholm, through developing an analytical tool for evaluating Emergency Medical Services (EMS) system design changes. The study also is an attempt to validate the proposed model used to generate the outcome measures for the study.

**Methods and results:**

This was done by combining a geographic information systems (GIS) simulation of driving times with register data on survival rates. The emergency resources comprised ambulance alone and ambulance plus fire services. The simulation model predicted a baseline survival rate of 3.9 per cent, and reducing the ambulance response time by one minute increased survival to 4.6 per cent. Adding the fire services as first responders (dual dispatch) increased survival to 6.2 per cent from the baseline level. The model predictions were validated using empirical data.

**Conclusion:**

We have presented an analytical tool that easily can be generalized to other regions or countries. The model can be used to predict outcomes of cardiac arrest prior to investment in EMS design changes that affect the alarm process, e.g. (1) static changes such as trimming the emergency call handling time or (2) dynamic changes such as location of emergency resources or which resources should carry a defibrillator.

## Background

Out-of-hospital cardiac arrest (OHCA) is a frequent and acute medical condition that requires immediate care [1,2]. Immediate treatment implies a survival chance of approximately 67 per cent, while the decline in survival rate without treatment is 5.5 per cent per minute and after 12 minutes a patient does not survive [3]. Resuscitation of OHCA victims can be improved with early alarm to emergency medical systems, early cardiopulmonary resuscitation (CPR), early defibrillation, early advanced care and post-resuscitation care, i.e. the ‘chain-of-survival’ concept [4,5]. Since survival is extremely time-sensitive, organisation of the emergency medical services (EMS) and other resources involved in the process is important.

Rational decisions about the organisation cannot be made without qualified estimations of the outcomes resulting from a specific intervention. In the present paper, we present an analytical tool for evaluating EMS system design changes and their impact on cardiac arrest outcomes. We evaluate marginal changes in the response time (defined as the interval between the OHCA incident and defibrillation) in the County of Stockholm, Sweden. Both extended and shortened response times were included as well as two alternative organisation forms of EMS defibrillation (ambulance alone and ambulance plus fire services). The latter form was useful for analysing the potential of alternative first-responders, which has been shown to improve the survival rate among patients with OHCA [6].

To estimate the survival rates, we use geographic information systems (GIS) simulation and combine it with register data on survival rates. GIS has previously been applied to locate potential areas of high and low rates of survival from OHCA [7,8], as well as to determine optimal locations for EMS resources [9]. As far as we know, this is the first study using GIS to analyse the association between location and organization of EMS resources and response times, which is directly related to survival. Alternatively, the association could be estimated by using logistic regressions on registered OHCA cases [10]. A limitation of the regression method is that it is bound to use the existing organisation of emergency resources.

We had a unique opportunity to validate the simulation results compared to published empirical data. As part of the Saving Lives in the Stockholm Area (SALSA) project, all fire stations in the County of Stockholm were first provided with automated external defibrillators (AEDs) and fire and rescue vehicles were then dispatched in parallel with ambulances to all OHCA cases [6]. The outcome of the SALSA project (16 additional survivors) showed good compliance with the predicted outcome (16 additional survivors). The results from the analytical tool can be used to support rational planning processes regarding interventions that affect the alarm process for OHCAs.

## Method

We used a general model to calculate the number of surviving OHCA patients in a specific region, pre-determined by the EMS system design and response strategies. ‘Surviving’ was defined as being alive 30 days after the OHCA, which in 94 per cent of the cases implies a successful neurological functioning for the survivors (Cerebral Performance Categories, CPC, score of 1–2) [11]. As the ‘chain-of-survival’ concept shows, the rate of survival depends on a number of factors. In this paper we keep all factors except time to defibrillation constant when calculating the changes in survival outcomes. However, the model was general in the sense that both static changes, such as trimming the emergency call handling time, and dynamic changes, such as location of emergency resources or which resources should carry a defibrillator (e.g. ambulance, fire services, police, lay persons) could be applied. The calculations can be summarised into three steps (the model is modified following Rauner & Bajmoczy [12]):

$$R_{i,j}=I_{i}\times \left(1-P_{A}\right)$$

where

1. The number of individuals in region *i* who suffer an OHCA and who can be saved by emergency resource *j* is given by

I_i_ = annual incidence of OHCA in region *i* (we follow Hollenberg et al. [6] and include the patients where some type of resuscitation measure was started)

P_A_ = probability that OHCA occurred after ambulance personnel arrival

In our case the region is the County of Stockholm and the emergency resources (*j*) comprise either ambulance alone or ambulance plus fire services.

2. The survival rate of patients resuscitated by emergency resource *j* in region *i* is given by a function of the time (*t*) from OHCA to defibrillation, i.e.

$$S_{i,j}=\sum_{t=1}^{N}\left(\frac{b_{i,t}}{{\text{POP}}_{i}}\right)\times {\mathrm{VF}}_{i,t}\times s_{i,t}$$

where

3. The number of patients surviving as a result of emergency resource *j* in region *i* is given by

$$\beta_{i,j}=R_{i,j}\times S_{i,j}$$

N = total number of time periods (minutes)

b_i,t_ = population in region *i* reached by emergency resource *j* at time *t*

POP_i_ = total population in region *i*

VF_i,t_ = probability in region *i* that a patient has ventricular fibrillation (VF) at time *t*

s_i,t_ = probability in region *i* that a patient having VF survives at time *t*

Finally, to obtain the marginal effects of an intervention, the procedure was repeated for the relevant alternatives and the difference in survival rates could thus be established.

## Data

**Figure 1:**
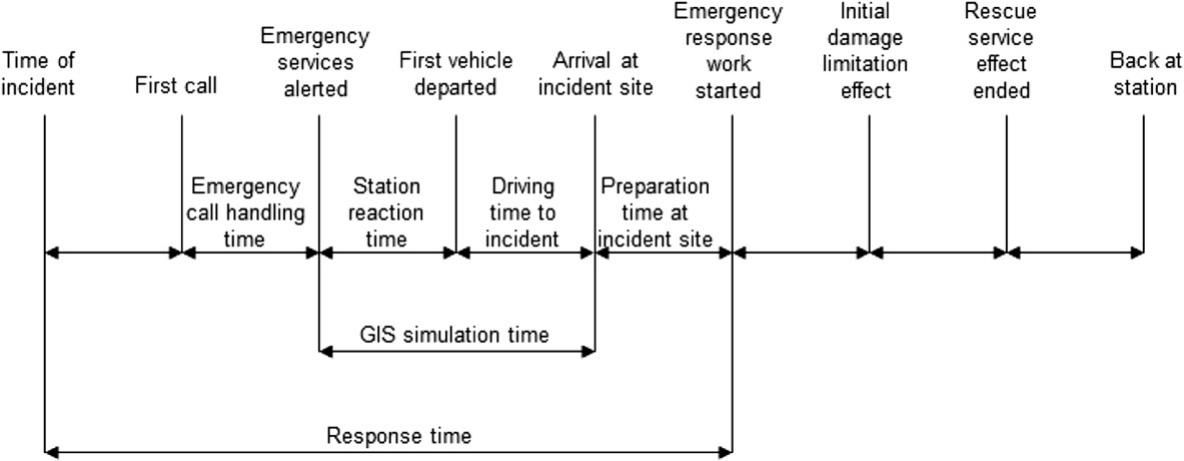
**The alarm process for emergency services from time of incident to back at station.** Notes: Modified from the Swedish Civil Contingencies Agency.

The geographic region chosen was the County of Stockholm, where the total population on 31 December 2007 was 1 949 516 (Statistics Sweden). In 2006 the incidence rate of patients with OHCA, where some type of resuscitation measure was started, was 816 and the probability that OHCA occurred after ambulance personnel arrival was 15 per cent [6]. Below, we report the conditions for the GIS simulations of the times from when emergency services are alerted to when the ambulance and fire services in the County of Stockholm arrive at the incident site. Figure 1 display the alarm process for emergency services. Also, information from the Swedish Cardiac Arrest Register (SCAR) was used to estimate the time-dependent probabilities that a patient has VF and that a patient with VF survives [13].

### GIS simulation of time from when emergency services are alerted to arrival at the incident site

The first step of our analytic model was to establish the gain in time from an OHCA to defibrillation achieved by dispatching fire services in parallel to ambulances. By using a geographic accessibility platform, PinPoint Sweden (PiPoS), developed by the Swedish Agency for Growth Policy Analysis, we arrived at the share of the population reached per minute by (1) ambulance, (2) fire services and (3) ambulance plus fire services. The simulation was performed by measuring the drive time plus the station reaction time from ambulance/fire stations to each person’s home in the County of Stockholm on 31 December 2007. The speed limits of the road network were assumed to be an approximation of the speed of the emergency vehicles. In localities, speed was assumed to be reduced by 20 per cent due to e.g. traffic congestion. ‘A locality consists of a group of buildings normally not more than 200 metres apart from each other, and must fulfil a minimum criterion of having at least 200 inhabitants’ [14]. Only the population in the County of Stockholm was considered, but no limitation for ambulance/fire services outside this area to be called out was set, i.e. a person suffering from an OHCA may be reached by an ambulance or fire services from a neighbouring county.

The station reaction time, i.e. the time from when the emergency services are alerted until the first vehicle departs, was estimated for ambulances but we have data for that time interval for fire services. The time was set to 90 seconds for all ambulance stations. Normally there is no stipulated station reaction time. Instead a ‘priority 1’ (highest priority) alarm is supposed to be responded to ‘immediately’. However, in practice and to achieve comparability with the fire services, 90 seconds is a good approximation and there is no reason to expect fire services and ambulance to have significantly different reaction times (Fredric Jonsson, Fire protection engineer, County of Jönköping, Sweden). For the fire services the station reaction time was more complicated, since there are both full-time and part-time stations. Full-time fire-fighters usually had a station reaction time of 90 seconds, while the time was longer and varied for part-time stations. Fortunately, the Swedish Rescue Services Agency had data on each fire station’s reaction time and these were included in the simulation.

To be able to perform the simulation, we also needed to assume a specific location where OHCAs occur. Most OHCAs, 65–74 per cent, occur in the person’s home [8,15,16]. We therefore used the inhabitants’ homes as the location of the OHCAs. The risk was assumed to be identical in all homes, i.e. we did not use any real data to identify actual cardiac arrest patient homes. Discussion of this limitation is included in the final section.

### The time from incident to start of emergency response work

The GIS simulation only displayed part of the alarm process. In addition to the station reaction time and the driving time to incident (time from emergency services alerted to arrival at incident site), which were simulated, we had to account for the process before and after this period of time in the case of an OHCA (see Figure 1). The alarm process after an OHCA starts with the time from incident to first call to the emergency call centre (in Sweden: 112). According to data from SCAR, this time was 2 minutes (median) in 2005–2006 in the County of Stockholm, which was shorter than the Swedish median time of 3 minutes [17]. Then the emergency call handling time starts. During this time, the operator handles the case and forwards it to the appropriate emergency services.

Following the emergency call handling time, the GIS-simulated time begins. Finally there is a time interval between arrival at the site and ‘hooking up’ the defibrillator to the patient (preparation time at incident site). With help from EMS experts in the SALSA project, we approximated both the emergency call handling time and the preparation time to be 1.5 minute on average. Now, summarising the total time from OHCA to defibrillation yields 2+1.5+GIS simulation time+1.5 minutes = 5 minutes plus GIS simulation time. Since the minimum station reaction time is 1.5 minutes, the shortest time from OHCA to defibrillation would be 5+1.5=6.5 minutes when driving time to incident is shorter than 1 minute. This implies that any cardiac arrest could not have treatment in < 6.5 minutes in the model.

### OHCA survival rates at various lengths of time from OHCA to defibrillation

Time is extremely important for successful treatment of OHCA and we used data from SCAR to estimate the probability of survival depending on the length of time from OHCA to defibrillation. A number of other factors also influenced the outcome, e.g. whether the OHCA was witnessed by a bystander, whether CPR was started prior to arrival of emergency services and the severity of the arrhythmia recorded by the first emergency responder [6]. These factors were kept constant, except for the severity of the arrhythmia, which depends on time. VF (including cases of pulseless ventricular tachycardia) is the only arrhythmia that can be effectively treated with defibrillation. More severe states of arrhythmia, i.e. asystole and pulseless electrical activity (PEA), are not influenced by a quicker defibrillation response. Admittedly, a quicker emergency response is likely to have a positive effect on patients with asystole or PEA as well, e.g. CPR can be performed while waiting for the ambulance. We did not include this effect in the analysis.

**Figure 2:**
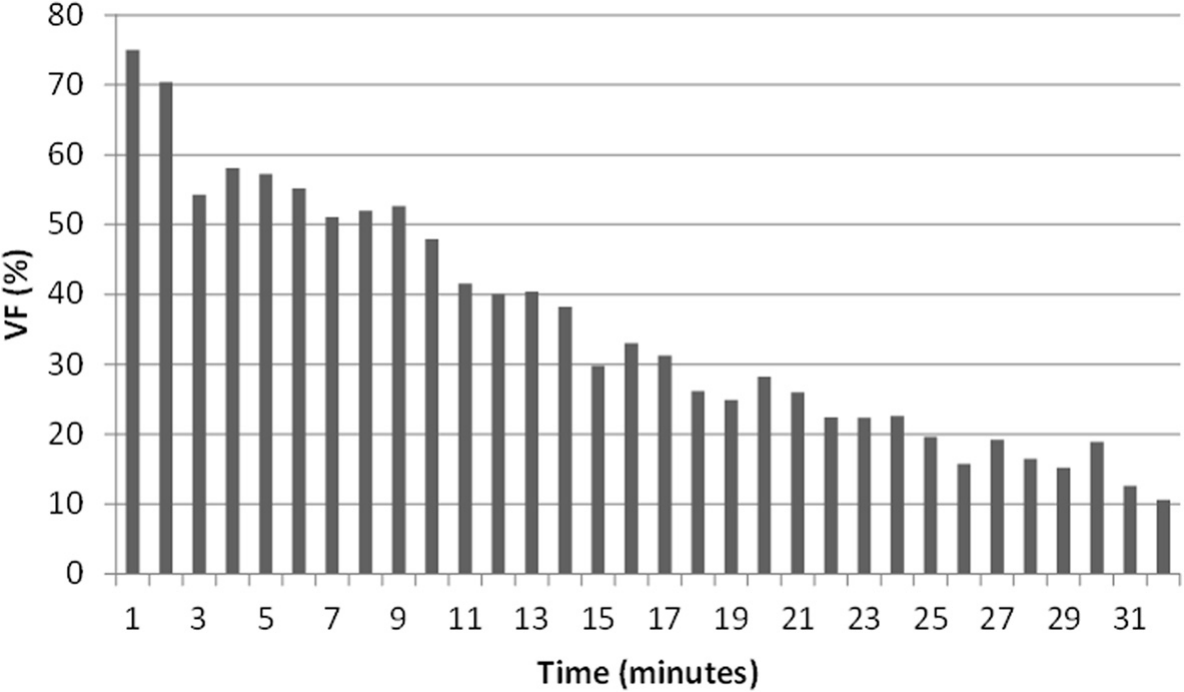
**Proportion of ventricular fibrillation (VF) on first electrocardiogram (ECG) in relation to time between OHCA and ECG.***Notes:From SCAR. The last proportion (31+) includes all cases above 31 minutes. Number of observations=16 360 (1990–2006).*

**Figure 3:**
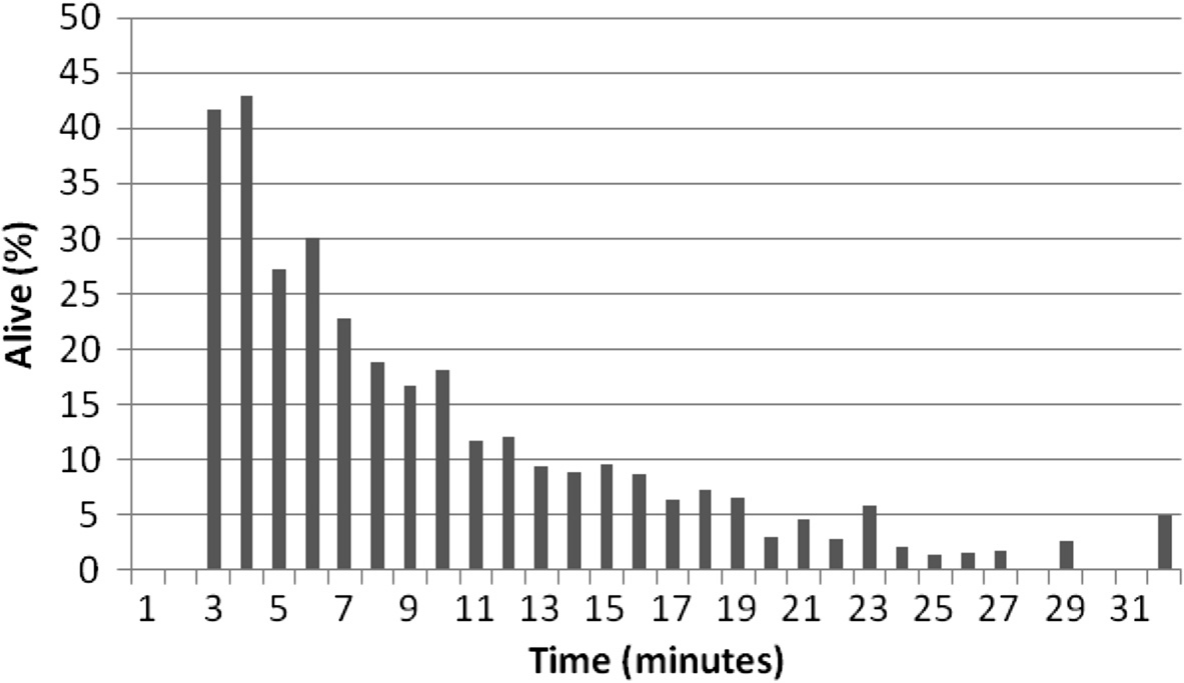
**Proportion of OHCA patients alive (1 month) with VF on first ECG in relation to time from OHCA to defibrillation.***Notes: From SCAR. The last proportion (31+) includes all cases above 31 minutes. Number of observations=6156 (1990–2006).*

Figure 2 shows how the proportion of VF depends on the time between an OHCA and the first electrocardiogram (ECG). The first time intervals represent OHCAs witnessed by EMS. This trend was included in our model and accounts for the decreasing possibilities of survival. Then, we continued to the next variable, which was survival rate of those with VF at a specific length of time from OHCA to defibrillation. Data from SCAR helped us estimate these proportions as well, and the pattern is presented in Figure 3. Since there are few observations of OHCA survivors above half an hour, the number of time periods (N) in the model is restricted to 31.

## Results

### Simulations and marginal effects of response time

**Figure 4:**
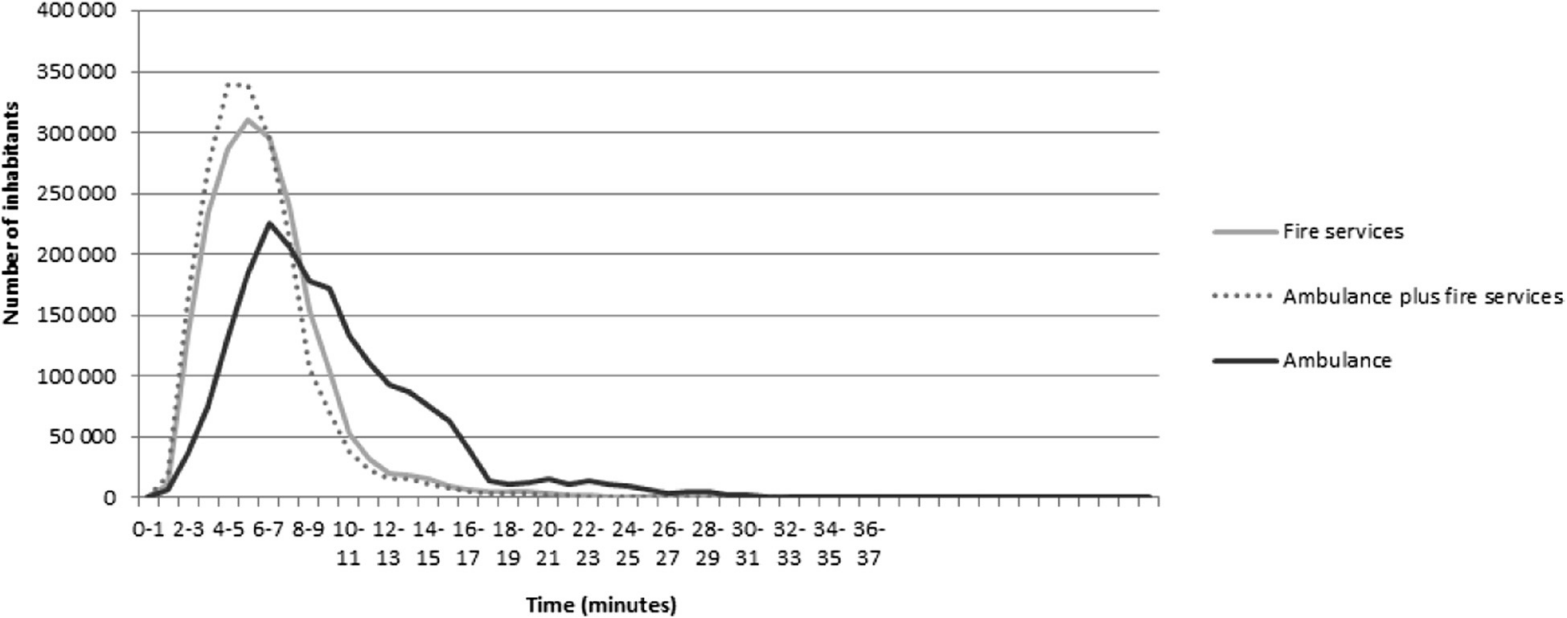
**Number of inhabitants of the County of Stockholm reached by emergency responder (fire services and/or ambulance) per time interval (minutes).** Notes: The data has to be adjusted by adding the remainder of the alarm process ( i.e. + 5 minutes) to capture the full picture.

Figure 4 shows the GIS simulations of how long it took from when the emergency services were alerted to the arrival at the incident site in the County of Stockholm. A large share of the inhabitants was reached within the time interval of 3–10 minutes. We recognize that the parallel dispatch of ambulances and fire services reached more inhabitants than the ambulance alone in shorter time intervals. Fire services alone came fairly close to the performance of parallel dispatch. Remember that the data displayed in Figure 4 has to be adjusted by adding the remainder of the alarm process, i.e. + 5 minutes in our case. Actually, this implies that the population reached will be zero for the first 6.5 minutes (5 minutes plus station reaction time).

**Figure 5:**
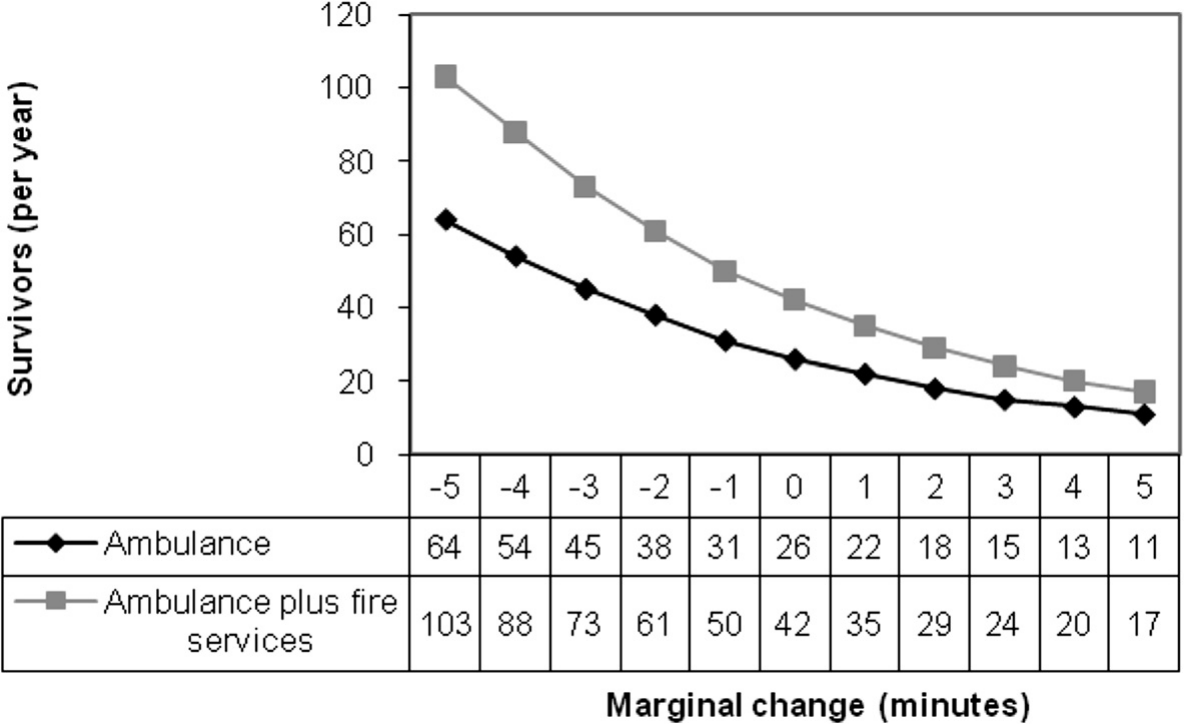
**Marginal effects in survivors (per year) of changing the time (in minutes) from time of incident to defibrillation.** Results for ambulance and ambulance plus fire services.

Based on the results from the simulations, we estimated the number of surviving patients (β). The baseline level of survivors per year was 26 (ambulance), or 3.9 per cent of the total number of OHCA patients. Engaging the fire services as first responders as well resulted in 42 survivors per year, implying that the number of additional lives saved by parallel dispatch of emergency resources was 16 per year. Changing the time from incidence to defibrillation affected the number of survivors (Figure 5). E.g. shortening/extending the emergency call handling time for the ambulance by 1 minute results in 31 (+5)/ 22 (−4) survivors per year. One further prediction of the analytic model is that the difference in number of survivors associated with parallel dispatch diminished the longer the time from incidence to defibrillation.

### Is the simulation model valid?

With all due respect for models, we were fortunate to be able to compare the modelled results of parallel dispatch with the ‘real’ outcome of the ‘Saving Lives in the Stockholm Area’ (SALSA) project. The results and a detailed description of the project are presented in Hollenberg et al. [6]. As part of the project, which was introduced in 2005, all 43 fire stations in the County of Stockholm were equipped with automated external defibrillators (AED) and fire services were dispatched in parallel with ambulances to all suspected cases of OHCA. The first emergency resource to arrive started CPR and attached an AED. Ambulances were dispatched in exactly the same manner as before and the length of time from incidence to defibrillation was only affected when the fire services were first on the scene.

The effects of the project were measured and evaluated during a pilot period from 1 December 2005 to 31 December 2006. A total of 863 patients with OHCA, where some type of resuscitation measure was started, were included and the proportion of patients alive after 1 month increased significantly from 4.4 to 6.8 per cent. Since the incidence of OHCA patients in the County of Stockholm was approximately 650–700 in 2006, the estimated number of additional lives saved by the project was 16 per year.

The analytic model predicted that 26 patients would survive when only an ambulance was dispatched and that 42 patients would survive when ambulance plus fire services were dispatched (baseline level). Thus, the simulation tells us that 16 extra patients would survive through an intervention of dual dispatch, which is also what ‘reality’ tells us. We believe that the compliance of the analytic model with the results of the ‘real’ situation (SALSA) validates our model, although there were a number of uncertainty factors in both cases.

A historical control of the baseline level of survival was also possible. The model predicted that approximately 3.9 per cent of the OHCA patients would survive (ambulance EMS only). Hollenberg et al. [6] estimated that 4.4 per cent would survive (2004), yet earlier studies have estimated survival rates of 2.3 per cent (2000) and 3.3 per cent (2000–2002) in the Stockholm area [5,18]. In summary, we believe that the model did comply fairly well with the baseline level.

The SALSA project has continued its dispatch of fire and rescue vehicles with ambulances. It has also expanded the first responders to include all police vehicles (approximately 120 more defibrillators). Unpublished results indicate that survival from OHCA has further increased since the pilot period.

## Discussion

We have presented a model that combines geographic information systems (GIS) simulations of the lengths of emergency services times from an out-of-hospital cardiac arrest (OHCA) incident to defibrillation with data on survival rates from the Swedish Cardiac Arrest Register (SCAR). Simulations of ambulance alone as well as ambulance plus fire services were utilised as emergency resources. The results can be used to support rational planning processes regarding interventions that affect the alarm process for OHCAs, e.g. by using economic evaluations [19]. When informed about the outcomes involved, the decision maker has the opportunity to make a more enlightened policy choice and select the least expensive way to achieve a specific objective.

Implementation of policies, e.g. changes in regions or dynamic changes such as location of emergency resources or which resources should carry a defibrillator, requires new GIS simulations of the type we showed for dual dispatch. Trimming of the static time intervals in the alarm process (alarm: 2 minutes; emergency call handling: 1.5 minutes; preparation: 1.5 minutes) is important and can be directly impacted by e.g. public education (to decrease time to first call) and optimizing call handling (to decrease time to emergency service first alert). Marginal effects of changes in these intervals on survival rates can be evaluated directly by our model.

The model has several limitations that could potentially bias the results. Among other things, it was assumed that all OHCAs occur in the patients’ homes and that the risk is identical in all homes, despite the fact that OHCAs have been shown to have definite time-geographic distribution patterns [8]. E.g. commercial and business areas are more clustered during the day than at night and demographic factors such as age also matter. It has also been shown that there is a lower prevalence of and survival from initial VF from arrests at home versus public settings [20]. This factor should be considered in further analysis, although the proportions of VF used in the model are general for all cases of OHCA. Inclusion of actual locations of OHCAs could be used to improve upon the specific geographic risk, but it is not certain that actual locations alone would improve the prediction of future OHCAs (compared to patients’ homes).

Also, although a flat rate reduction of the driving speed in localities was included in the model, we are uncertain whether it correctly captures variations in the driving speed of emergency vehicles. Traffic congestion, road works and the choices of route may be factors that complicate the simulation. The model does not take vertical distances, e.g. floors above street level, into account, which naturally leads to further delays in arrival at patients’ side. Actual call-to-on-scene times for real OHCA events could be used to validate the speed limit based numbers as well as the assumption that the response teams are always in their stations.

Moreover, there are uncertainties regarding the estimations of the static time intervals, e.g. the emergency call handling time. In our model the emergency call handling time is estimated to be 1.5 minutes on average, yet according to SOS Alarm AB, the company responsible for handling 112 emergency calls and coordinating rescue work, it might be as long as 4 minutes on average for ‘priority 1’ ambulance calls (Mikael Björkander, SOS Alarm AB, e-mail 23 October 2008). If so, there certainly exists potential to improve this time interval. Using SOS Alarm AB’s figure of 4 minutes, the simulation model yields the baseline numbers of survivors of 15 (ambulance) and 24 (ambulance plus fire services); the baseline level of survivors is as low as 2.2% and the additional lives saved through dual dispatch is 9 instead of 16 per year. One minute shorter emergency call handling time would yield a large benefit to the OHCA population, +3 survivors (ambulance) and +5 survivors (ambulance plus fire services).

It is clear that a sensitivity analysis of the results is necessary to provide a good basis for decisions. One further opportunity for improvement is to address variability in the model. We used point estimates for all components of the model. Variability around each of these estimates can have a compounding effect and ultimately affect the accuracy of the model. Assessment of the effects of errors in assumptions for values of variables used in the model should be addressed in some way, either internalized in the model or as a sensitivity analysis of the outcome e.g. [19].

With the limitations, the simulation results for dual dispatch comply well with the results from a ‘real life’ intervention. In addition, Pell et al. (2001) estimated that a reduced ambulance response time from arrival at the scene within 14 minutes in 90 per cent of all emergency calls to arrival within 8 minutes would increase the survival rate from 6 to 8 per cent; reducing the response time to 5 minutes would increase the survival rate to 10–11 per cent [10]. Even if these results are not directly comparable with ours, they are in the same order of magnitude.

Although the analytic model was calibrated for the County of Stockholm, it can be generalised to other Swedish counties or regions, as well as other countries. The data that is needed to calculate the number of patients surviving as a result of a specific EMS design change in a region is very general and easily available (see Section 2). By adjusting the model specification for *r* (number of patients) and *s* (survival rate) we may calculate effects of other changes as well, e.g. the effect of earlier CPR or the effect of faster response to other diagnoses than OHCA. Before analysing the impact of the EMS design changes on other diagnoses, we need to establish a relation between survival rates as a function of time depending on the intervention considered.

The geographic location of defibrillators is possibly the most interesting factor to elaborate on and it also affects the marginal benefits of static response time changes, e.g. the increase in number of survivors was larger after introducing dual dispatch. When we consider geographic placement, the model is useful for analysing the effect of defibrillator placement in emergency vehicles (or at least professionals in moving vehicles, e.g. taxis, security personnel, home care staff). One natural step to further increase OHCA survival would be to introduce public access defibrillation (PAD), i.e. to place AEDs in the community for use by laypersons. The analytic model does not apply to this setting with stationary AEDs. However, although evaluations of PAD trials have shown a positive impact of survival, the cost-effectiveness of these programs remains uncertain. This is due to the large resources involved and the limited potential to save lives (less than 5 percent of OHCA occur in large public buildings) [21-23].

In summary, despite the complexity of modelling an intervention of this type, we believe that the results at hand are useful for deploying effective EMS system design strategies. The possibility of testing where defibrillators should be placed geographically is deemed particularly useful and focuses on first responder defibrillation, which has potential to have an effect in private homes where the vast majority of OHCAs happen [23]. Extension of the tool to analyse changes in e.g. the proportion where CPR was started prior to arrival of emergency services is possible. More applications using GIS technology in time-sensitive emergency conditions would be equally interesting.

## Competing interests

The author declare that he have no competing interest.
